# [2-(4-Methylbenzoyl)phenyl](4-methylphenyl)methanone

**DOI:** 10.1107/S1600536811028820

**Published:** 2011-07-23

**Authors:** P. Narayanan, K. Sethusankar, Meganathan Nandakumar, Arasambattu K. Mohanakrishnan

**Affiliations:** a Department of Physics, RKM Vivekananda College (Autonomous), Chennai 600 004, India; b Department of Organic Chemistry, University of Madras, Guindy Campus, Chennai 600 025, India

## Abstract

The asymmetric unit of the title compound, C_22_H_18_O_2_, contains one half-mol­ecule, the complete mol­ecule being generated by the operation of a crystallographic twofold rotation axis. The carbonyl group and the two C atoms attached to it forms inter­planar angles of 23.67 (7)° with the methyl-substituted phenyl ring and 50.74 (8)° with the central ring. In the crystal, mol­ecules are linked into infinite chains along the *b*-axis direction by inter­molecular C—H⋯O inter­actions, generating *R* ^2^_2_(10) graph-set motifs.

## Related literature

For the uses and biological importance of diketones, see: Bennett *et al.* (1999 ▶); Sato *et al.* (2008 ▶). For related structures, see: Muto *et al.* (2010 ▶); Khan *et al.* (2009 ▶); For asymmetry parameters, see: Nardelli (1983 ▶); Macrae *et al.* (2008 ▶). For graph-set notation: Bernstein *et al.* (1995 ▶).
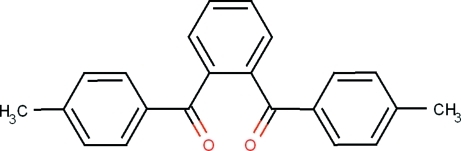


## Experimental

### 

#### Crystal data

C_22_H_18_O_2_
*M* *_r_* = 314.36
Monoclinic, 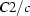

*a* = 20.7432 (13) Å
*b* = 7.7564 (4) Å
*c* = 11.3946 (6) Å
β = 114.314 (5)°
*V* = 1670.70 (17) Å^3^
*Z* = 4
Mo *K*α radiation
μ = 0.08 mm^−1^
*T* = 295 K
0.30 × 0.25 × 0.20 mm

#### Data collection

Bruker Kappa APEXII CCD diffractometer
Absorption correction: multi-scan (*SADABS*; Sheldrick, 1996 ▶) *T*_min_ = 0.977, *T*_max_ = 0.984
17689 measured reflections
2133 independent reflections
1729 reflections with *I* > 2σ(*I*)
*R*_int_ = 0.076

#### Refinement

*R*[*F* ^2^ > 2σ(*F* ^2^)] = 0.050
*wR*(*F* ^2^) = 0.154
*S* = 1.03
2133 reflections
110 parameters
H-atom parameters constrained
Δρ_max_ = 0.30 e Å^−3^
Δρ_min_ = −0.28 e Å^−3^

### 

Data collection: *APEX2* (Bruker, 2004 ▶); cell refinement: *SAINT* (Bruker, 2004 ▶); data reduction: *SAINT*; program(s) used to solve structure: *SHELXS97* (Sheldrick, 2008 ▶); program(s) used to refine structure: *SHELXL97* (Sheldrick, 2008 ▶); molecular graphics: *ORTEP-3* (Farrugia, 1997 ▶) and *Mercury* (Macrae *et al.*, 2008 ▶); software used to prepare material for publication: *SHELXL97* and *PLATON* (Spek, 2009) ▶.

## Supplementary Material

Crystal structure: contains datablock(s) global, I. DOI: 10.1107/S1600536811028820/rk2284sup1.cif

Structure factors: contains datablock(s) I. DOI: 10.1107/S1600536811028820/rk2284Isup2.hkl

Supplementary material file. DOI: 10.1107/S1600536811028820/rk2284Isup3.cml

Additional supplementary materials:  crystallographic information; 3D view; checkCIF report

## Figures and Tables

**Table 1:** Hydrogen-bond geometry (Å, °)

*D*—H⋯*A*	*D*—H	H⋯*A*	*D*⋯*A*	*D*—H⋯*A*
C1—H1⋯O1^i^	0.93	2.62	3.4305 (17)	145

Symmetry code: (i) 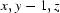.

**Fig. 1.**
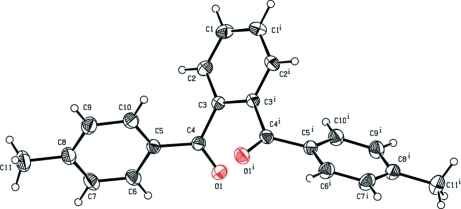
The molecular structure of the title compound with the atom numbering scheme. Displacement ellipsoids are shown at 30% probability level. The H atoms are presented as a small spheres of arbitrary radius. Related atoms have symmetry code: (i) -x+1, y, -z+3/2.

**Fig. 2.**
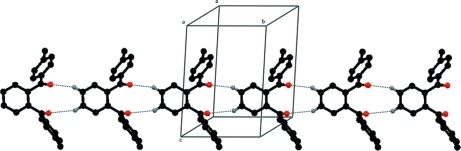
The crystal packing of the title compound, viewed down c axis, showing molecules linked along b axis. Intermolecular hydrogen bonds are shown in as dashed lines.

## supplementary crystallographic information

### Comment

Diketones are popular in organic synthesis, for their applications in biology and medicine. They are known to exhibit antioxidants, antitumour and antibacterial activities (Bennett *et al.*, 1999). They are also key intermediates in the preparation of various heterocyclic compounds (Sato *et al.*, 2008).

The title compound C_22_H_18_O_2_, contains one half molecule in the asymmetric unit, the complete molecule being generated by twofold rotation, with direction [0 1 0], having symmetry code: (i) *-x*+1, *y*, *-z*+3/2. X-ray analysis confirms the molecular structure and atom connectivity of the compound as illustrated in (Fig. 1). The carbonyl group (C3/C4/C5/O1) forms an interplanar angle of 23.67 (7)° with the phenyl ring (C5/C6/C7/C8/C9/C10). The deviation of atom O1 from the phenyl ring (C5/C6/C7/C8/C9/C10) is -0.4719 (19)Å (Nardelli, 1983). The title compound exhibits structural similarities with the already reported related structures (Muto *et al.*, 2010; Khan *et al.*, 2009).

The central phenyl ring (C1/C2/C3/C1^i^/C2^i^/C3^i^) forms dihedral angles of 67.14 (17)° and 50.74 (8)° with the phenyl ring (C5/C6/C7/C8/C9/C10) and the mean plane of the carbonyl group (C3/C4/C5/O1), respectively. The dihedral angle between the phenyl rings (C5/C6/C7/C8/C9/C10) and (C5^i^/C6^i^/C7^i^/C8^i^/C9^i^/C10^i^) is 82.83 (2)° (Macrae *et al.*, 2008), and thus they are almost orthogonal to each other.

The crystal packing is stabilized by C—H···O intermolecular interactions. The molecules are linked into infinite chains along the *b* axis *via* C1—H1···O1^ii^ hydrogen bonds, generating the *R*^2^_2_(10) graphset motifs (Bernstein *et al.*, 1995). The symmetry code: (ii) *x*, -1+*y*, *z* (look Table 1). The packing view of the compound is shown in (Fig. 2).

### Experimental

To a stirred suspension of benzo[*c*]furan, 1,3-bis(4-methylphenyl)-4,7-dihydro-2-benzofuran (3 g, 9.554 mmol) in dry *THF* (20 ml), lead tetra acetate (4.23 g, 9.5 mmol) was added and refluxed at 343 K for half an hour. The reaction mixture was then poured into water (200 ml) and extracted with ethyl acetate (2 x 20 ml), washed with brine solution and dried (Na_2_SO_4_). The removal of solvent *in vacuo* followed by crystallization from methanol afforded the title compound, (4-methylphenyl){2-[(4-methylphenyl)carbonyl]phenyl}methanone as a colourless solid.

### Refinement

The hydrogen atoms were placed in calculated positions with C—H = 0.93–0.96Å and refined in the riding model with fixed isotropic displacement parameters: *U*_iso_(H) = 1.5*U*_eq_(C) for methyl atoms and *U*_iso_(H) = 1.2*U*_eq_(C) for the aryl atoms.

### Crystal data

**Table:**

C_22_H_18_O_2_	*F*(000) = 664
*M**_r_* = 314.36	*D*_x_ = 1.250 Mg m^−^^3^
Monoclinic, *C*2/*c*	Mo *K*α radiation, λ = 0.71073 Å
Hall symbol: -C 2yc	Cell parameters from 2133 reflections
*a* = 20.7432 (13) Å	θ = 2.2–28.6°
*b* = 7.7564 (4) Å	µ = 0.08 mm^−^^1^
*c* = 11.3946 (6) Å	*T* = 295 K
β = 114.314 (5)°	Block, colourless
*V* = 1670.70 (17) Å^3^	0.30 × 0.25 × 0.20 mm
*Z* = 4	

### Data collection

**Table:**

Bruker Kappa APEXII CCD diffractometer	2133 independent reflections
Radiation source: fine-focus sealed tube	1729 reflections with *I* > 2σ(*I*)
graphite	*R*_int_ = 0.076
ω–scans	θ_max_ = 28.6°, θ_min_ = 2.2°
Absorption correction: multi-scan (*SADABS*; Sheldrick, 1996)	*h* = −27→27
*T*_min_ = 0.977, *T*_max_ = 0.984	*k* = −10→10
17689 measured reflections	*l* = −15→15

### Refinement

**Table:**

Refinement on *F*^2^	Primary atom site location: structure-invariant direct methods
Least-squares matrix: full	Secondary atom site location: difference Fourier map
*R*[*F*^2^ > 2σ(*F*^2^)] = 0.050	Hydrogen site location: inferred from neighbouring sites
*wR*(*F*^2^) = 0.154	H-atom parameters constrained
*S* = 1.03	*w* = 1/[σ^2^(*F*_o_^2^) + (0.0914*P*)^2^ + 0.4904*P*] where *P* = (*F*_o_^2^ + 2*F*_c_^2^)/3
2133 reflections	(Δ/σ)_max_ = 0.005
110 parameters	Δρ_max_ = 0.30 e Å^−^^3^
0 restraints	Δρ_min_ = −0.28 e Å^−^^3^

### Special details

**Table:**

Geometry. All s.u.'s (except the s.u. in the dihedral angle between two l.s. planes) are estimated using the full covariance matrix. The cell s.u.'s are taken into account individually in the estimation of s.u.'s in distances, angles and torsion angles; correlations between s.u.'s in cell parameters are only used when they are defined by crystal symmetry. An approximate (isotropic) treatment of cell s.u.'s is used for estimating s.u.'s involving l.s. planes.
Refinement. Refinement of *F*^2^ against ALL reflections. The weighted *R*-factor *wR* and goodness of fit *S* are based on *F*^2^, conventional *R*-factors *R* are based on *F*, with *F* set to zero for negative *F*^2^. The threshold expression of *F*^2^ > σ(*F*^2^) is used only for calculating *R*-factors(gt) *etc*. and is not relevant to the choice of reflections for refinement. *R*-factors based on *F*^2^ are statistically about twice as large as those based on *F*, and *R*-factors based on ALL data will be even larger.

### Fractional atomic coordinates and isotropic or equivalent isotropic displacement parameters (Å^2^)

**Table:**

	*x*	*y*	*z*	*U*_iso_*/*U*_eq_	
C1	0.47990 (8)	−0.45596 (18)	0.68469 (16)	0.0577 (4)	
H1	0.4669	−0.5598	0.6403	0.069*	
C2	0.45881 (7)	−0.30216 (17)	0.61934 (13)	0.0493 (3)	
H2	0.4310	−0.3030	0.5312	0.059*	
C3	0.47868 (6)	−0.14603 (15)	0.68400 (11)	0.0375 (3)	
C4	0.46029 (6)	0.01963 (15)	0.61000 (11)	0.0380 (3)	
C5	0.38557 (6)	0.05007 (15)	0.52013 (11)	0.0384 (3)	
C6	0.37060 (7)	0.16656 (18)	0.41976 (13)	0.0478 (3)	
H6	0.4074	0.2221	0.4083	0.057*	
C7	0.30152 (8)	0.2002 (2)	0.33701 (13)	0.0535 (4)	
H7	0.2924	0.2766	0.2691	0.064*	
C8	0.24545 (7)	0.12297 (18)	0.35264 (13)	0.0508 (4)	
C9	0.26051 (7)	0.0089 (2)	0.45387 (14)	0.0529 (4)	
H9	0.2236	−0.0433	0.4668	0.063*	
C10	0.32965 (7)	−0.02856 (18)	0.53609 (13)	0.0474 (3)	
H10	0.3387	−0.1072	0.6027	0.057*	
C11	0.17032 (9)	0.1623 (3)	0.26113 (19)	0.0739 (5)	
H11A	0.1696	0.2622	0.2109	0.111*	
H11B	0.1425	0.1841	0.3093	0.111*	
H11C	0.1509	0.0656	0.2050	0.111*	
O1	0.50637 (5)	0.12345 (12)	0.62240 (9)	0.0503 (3)	

### Atomic displacement parameters (Å^2^)

**Table:**

	*U*^11^	*U*^22^	*U*^33^	*U*^12^	*U*^13^	*U*^23^
C1	0.0579 (8)	0.0333 (6)	0.0742 (10)	−0.0053 (6)	0.0195 (7)	−0.0101 (6)
C2	0.0496 (7)	0.0405 (7)	0.0470 (7)	−0.0063 (5)	0.0091 (6)	−0.0084 (5)
C3	0.0360 (5)	0.0337 (6)	0.0368 (6)	−0.0015 (4)	0.0088 (5)	−0.0008 (4)
C4	0.0414 (6)	0.0359 (6)	0.0318 (6)	−0.0023 (4)	0.0100 (5)	−0.0016 (4)
C5	0.0405 (6)	0.0371 (6)	0.0324 (6)	0.0011 (4)	0.0097 (5)	−0.0003 (4)
C6	0.0487 (7)	0.0482 (7)	0.0442 (7)	0.0033 (5)	0.0167 (6)	0.0096 (5)
C7	0.0557 (8)	0.0531 (8)	0.0437 (7)	0.0116 (6)	0.0125 (6)	0.0121 (6)
C8	0.0450 (7)	0.0493 (7)	0.0471 (7)	0.0084 (6)	0.0079 (6)	−0.0061 (5)
C9	0.0418 (7)	0.0575 (8)	0.0566 (8)	−0.0040 (6)	0.0176 (6)	−0.0025 (6)
C10	0.0484 (7)	0.0482 (7)	0.0416 (7)	−0.0025 (5)	0.0143 (5)	0.0049 (5)
C11	0.0483 (8)	0.0751 (11)	0.0757 (11)	0.0150 (7)	0.0028 (8)	−0.0027 (9)
O1	0.0479 (5)	0.0442 (5)	0.0487 (5)	−0.0100 (4)	0.0098 (4)	0.0032 (4)

### Geometric parameters (Å, °)

**Table:**

C1—C1^i^	1.374 (3)	C6—H6	0.9300
C1—C2	1.379 (2)	C7—C8	1.383 (2)
C1—H1	0.9300	C7—H7	0.9300
C2—C3	1.3890 (16)	C8—C9	1.384 (2)
C2—H2	0.9300	C8—C11	1.507 (2)
C3—C3^i^	1.396 (2)	C9—C10	1.383 (2)
C3—C4	1.4975 (16)	C9—H9	0.9300
C4—O1	1.2128 (15)	C10—H10	0.9300
C4—C5	1.4832 (16)	C11—H11A	0.9600
C5—C10	1.3872 (18)	C11—H11B	0.9600
C5—C6	1.3887 (17)	C11—H11C	0.9600
C6—C7	1.3778 (19)		
			
C1^i^—C1—C2	120.07 (8)	C6—C7—C8	121.50 (13)
C1^i^—C1—H1	120.0	C6—C7—H7	119.2
C2—C1—H1	120.0	C8—C7—H7	119.2
C1—C2—C3	120.60 (12)	C7—C8—C9	118.11 (12)
C1—C2—H2	119.7	C7—C8—C11	120.53 (14)
C3—C2—H2	119.7	C9—C8—C11	121.36 (15)
C2—C3—C3^i^	119.31 (7)	C10—C9—C8	120.95 (13)
C2—C3—C4	119.89 (10)	C10—C9—H9	119.5
C3^i^—C3—C4	120.54 (6)	C8—C9—H9	119.5
O1—C4—C5	121.61 (11)	C9—C10—C5	120.58 (12)
O1—C4—C3	119.89 (10)	C9—C10—H10	119.7
C5—C4—C3	118.48 (10)	C5—C10—H10	119.7
C10—C5—C6	118.59 (11)	C8—C11—H11A	109.5
C10—C5—C4	122.14 (11)	C8—C11—H11B	109.5
C6—C5—C4	119.21 (11)	H11A—C11—H11B	109.5
C7—C6—C5	120.25 (13)	C8—C11—H11C	109.5
C7—C6—H6	119.9	H11A—C11—H11C	109.5
C5—C6—H6	119.9	H11B—C11—H11C	109.5
			
C1^i^—C1—C2—C3	1.0 (3)	C10—C5—C6—C7	−1.1 (2)
C1—C2—C3—C3^i^	1.0 (2)	C4—C5—C6—C7	−178.36 (12)
C1—C2—C3—C4	175.06 (13)	C5—C6—C7—C8	1.4 (2)
C2—C3—C4—O1	−125.83 (13)	C6—C7—C8—C9	−0.5 (2)
C3^i^—C3—C4—O1	48.2 (2)	C6—C7—C8—C11	179.79 (14)
C2—C3—C4—C5	52.62 (16)	C7—C8—C9—C10	−0.8 (2)
C3^i^—C3—C4—C5	−133.37 (15)	C11—C8—C9—C10	178.91 (14)
O1—C4—C5—C10	−156.10 (13)	C8—C9—C10—C5	1.2 (2)
C3—C4—C5—C10	25.47 (17)	C6—C5—C10—C9	−0.2 (2)
O1—C4—C5—C6	21.08 (18)	C4—C5—C10—C9	177.00 (12)
C3—C4—C5—C6	−157.35 (12)		

Symmetry codes: (i) −*x*+1, *y*, −*z*+3/2.

### Hydrogen-bond geometry (Å, °)

**Table:**

*D*—H···*A*	*D*—H	H···*A*	*D*···*A*	*D*—H···*A*
C1—H1···O1^ii^	0.93	2.62	3.4305 (17)	145.

Symmetry codes: (ii) *x*, *y*−1, *z*.

## References

[bb1] Bennett, I., Broom, N. J. P., Cassels, R., Elder, J. S., Masson, N. D. & O’Hanlon, P. J. (1999). *Bioorg. Med. Chem. Lett.* **9**, 1847–1852. 10.1016/s0960-894x(99)00296-6 10406653

[bb2] Bernstein, J., Davis, R. E., Shimoni, L. & Chang, N. L. (1995). *Angew. Chem. Int. Ed. Engl.* **34**, 1555–1573.

[bb3] Bruker (2004). *APEX2* and *SAINT* Bruker AXS Inc., Madison, Wisconsin, USA.

[bb4] Farrugia, L. J. (1997). *J. Appl. Cryst.* **30**, 565.

[bb5] Khan, F. N., Manivel, P., Prabakaran, K., Hathwar, V. R. & Ng, S. W. (2009). *Acta Cryst.* E**65**, o2745. 10.1107/S1600536809041270 PMC2971431 21578340

[bb6] Macrae, C. F., Bruno, I. J., Chisholm, J. A., Edgington, P. R., McCabe, P., Pidcock, E., Rodriguez-Monge, L., Taylor, R., van de Streek, J. & Wood, P. A. (2008). *J. Appl. Cryst.* **41**, 466–470.

[bb7] Muto, T., Kato, Y., Nagasawa, A., Okamoto, A. & Yonezawa, N. (2010). *Acta Cryst.* E**66**, o2752. 10.1107/S1600536810039620 PMC3009178 21588956

[bb8] Nardelli, M. (1983). *Acta Cryst.* C**39**, 1141–1142.

[bb9] Sato, K., Yamazoe, S., Yamamoto, R., Ohata, S., Tarui, A., Omote, M., Kumadaki, I. & Ando, A. (2008). *Org. Lett.* **10**, 2405–2408. 10.1021/ol800660y 18476711

[bb10] Sheldrick, G. M. (1996). *SADABS* University of Göttingen, Germany.

[bb11] Sheldrick, G. M. (2008). *Acta Cryst.* A**64**, 112–122. 10.1107/S0108767307043930 18156677

[bb12] Spek, A. L. (2009). *Acta Cryst* D**65**, 148–155. 10.1107/S090744490804362X PMC2631630 19171970

